# Role of ARHGEF3 as a GEF and mTORC2 Regulator

**DOI:** 10.3389/fcell.2021.806258

**Published:** 2022-01-31

**Authors:** Sana Abdul Khaliq, Zobia Umair, Mee-Sup Yoon

**Affiliations:** ^1^ Department of Molecular Medicine, Gachon University College of Medicine, Incheon, South Korea; ^2^ Department of Health Sciences and Technology, GAIHST, Gachon University, Incheon, South Korea; ^3^ Lee Gil Ya Cancer and Diabetes Institute, Gachon University, Incheon, South Korea

**Keywords:** ARHGEF3, XPLN, rho guanine nucleotide exchange factors, mTORC2, Akt

## Abstract

Guanine nucleotide exchange factors (GEFs) activate GTPases by stimulating the release of guanosine diphosphate to permit the binding of guanosine triphosphate. ARHGEF3 or XPLN (exchange factor found in platelets, leukemic, and neuronal tissues) is a selective guanine nucleotide exchange factor for Rho GTPases (RhoGEFs) that activates RhoA and RhoB but not RhoC, RhoG, Rac1, or Cdc42. ARHGEF3 contains the diffuse B-cell lymphoma homology and pleckstrin homology domains but lacks similarity with other known functional domains. ARHGEF3 also binds the mammalian target of rapamycin complex 2 (mTORC2) and subsequently inhibits mTORC2 and Akt. *In vivo* investigation has also indicated the communication between ARHGEF3 and autophagy-related muscle pathologies. Moreover, studies on genetic variation in ARHGEF3 and genome-wide association studies have predicted exciting novel roles of ARHGEF3 in controlling bone mineral density, platelet formation and differentiation, and Hirschsprung disease. In conclusion, we hypothesized that additional biochemical and functional studies are required to elucidate the detailed mechanism of ARHGEF3-related pathologies and therapeutics.

## 1 Introduction

ARHGEF3 (or XPLN, exchange factor found in platelets, leukemic, and neuronal tissues) was identified as RhoGEF (Rho guanine nucleotide exchange factor) for Rho GTPases through an expressed sequence tag) database search, using the diffuse B-cell lymphoma (Dbl) homology (DH) domain query in the BLASTN system (Thiesen et al., 2000). It selectively activates RhoA and RhoB (Arthur et al., 2002). Notably, the noncanonical role of ARHGEF3 has been presented; ARHGEF3 binds the mammalian target of rapamycin complex 2 (mTORC2), inhibiting mTORC2 kinase activity, primarily for Akt.

This review focuses on ARHGEF3, providing an overview of its functions under various physiological conditions. We will address its structure and function as a GEF and mTORC2 regulator. According to *in vivo* research, ARHGEF3 and autophagy-related muscle diseases are linked. Furthermore, studies on ARHGEF3 genetic variation and genome-wide association studies (GWASs) have identified interesting new roles of ARHGEF3 in modulating bone mineral density (BMD), platelet formation and differentiation, and Hirschsprung disease. The present review provides a complete understanding of the intricacies of genetic association networks engaged in ARHGEF3-related pathologies, consequently prompting advancements in potential treatment modalities via mechanistic investigations.

## 2 Characteristics of ARHGEF3

### 2.1 ARHGEF3 Belongs to the Dbl Family of GEFs

**FIGURE 1:**
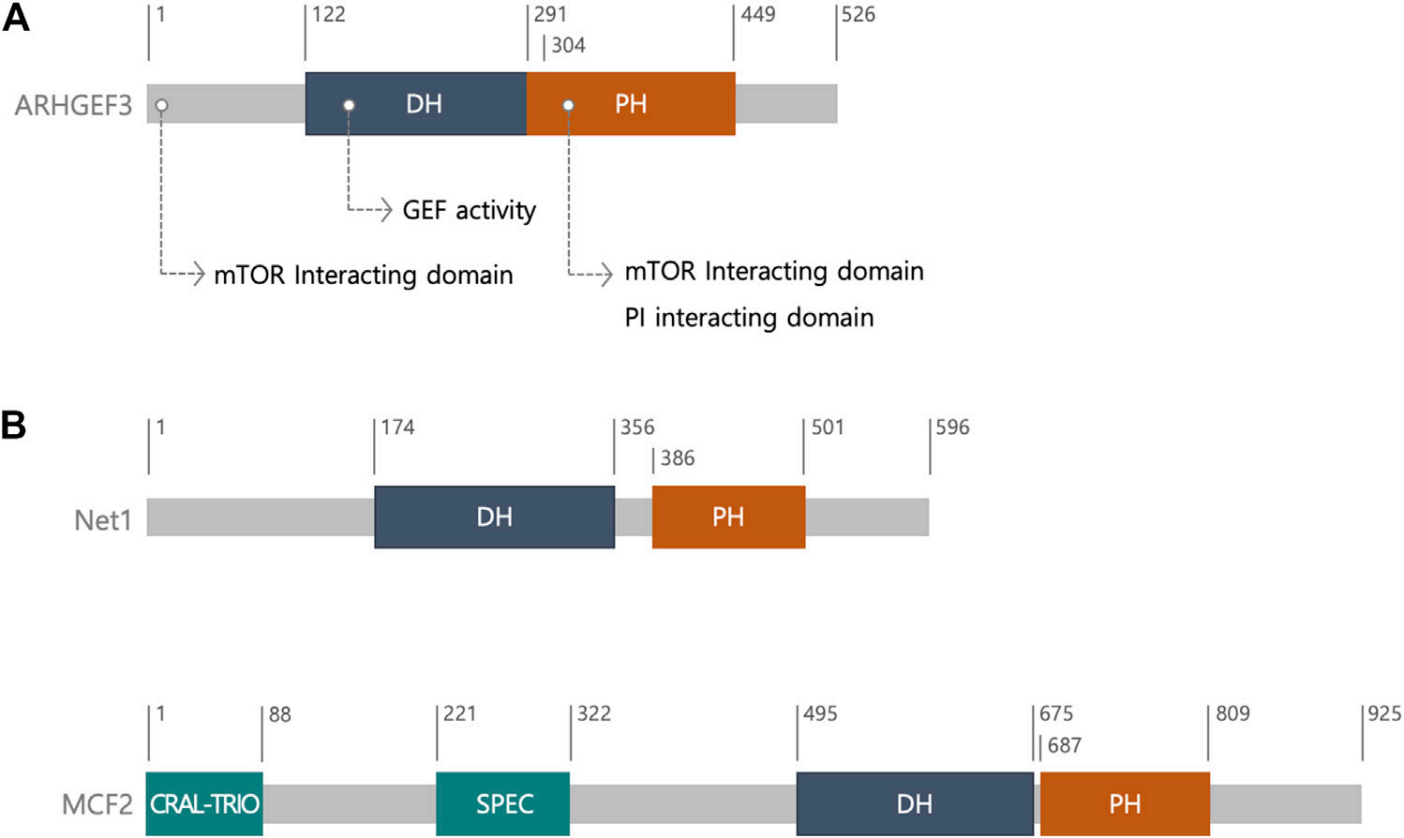
The structure of ARHGEF3. **(A)** Schematic diagram and ARHGEF3 with its function. **(B)** Schematic diagram of neuroepithelial cell transforming gene 1 (Net1) and MCF2. ARHGEF3’s DH-PH domains have a sequence similarity of over 69 percent to that of Net1. MCF2 is one of the diffuse B-cell lymphoma (Dbl) family. The CRAL-TRIO domain is involved in interaction with inositol phospholipids and SPEC domain promotes the interaction with membrane proteins. Abbreviation; the diffuse B-cell lymphoma (Dbl) homology (DH); pleckstrin homology (PH); cellular retinaldehyde-binding protein (CRALBP) and TRIO guanine exchange factor (CRAL-TRIO); spectrin (SPEC).

In humans, ARHGEF3 consists of 526 amino acids, including a DH domain and a pleckstrin homology (PH) domain in tandem (Figure 1) (Rossman et al., 2005; Murayama et al., 2012). The DH-PH domains of ARHGEF3 share over 69% sequence similarity with those of neuroepithelial cell transforming gene 1 (Net1), which belongs to the diffuse B-cell lymphoma (Dbl) family (Murayama et al., 2012). The Dbl gene family, with 70 members in humans, and the DOCK (dedicator of cytokinesis) gene family, with 11 members, roughly encode 81 RhoGEF genes in the human genome members (Meller et al., 2005; Rossman et al., 2005; Cook et al., 2014). The Dbl family is characterized by a DH catalytic domain, followed by an adjacent PH domain, C-terminal to the DH domain (Rossman et al., 2005) (Figure 1). In most cases, together they provide the minimal structural units required to catalyze the exchange reaction of guanosine diphosphate (GDP) for guanosine triphosphate (GTP) (Rossman et al., 2005). In most Dbl RhoGEFs, the PH domain helps the intrinsic catalytic activity of RhoGEFs by regulating their membrane localization through phospholipid binding, and the allosteric regulation of RhoGEFs via their association with other proteins (Müller et al., 2020; Singh et al., 2021). In contrast, the DOCK family of GEFs are structurally and mechanistically different from the Dbl family and have two characteristic domains, the DOCK homology region 1 (a phospholipid-binding domain that can recruit the GEFs to the membrane) and DHR2 (a catalytic domain) (Cote et al., 2005; Meller et al., 2005). DHR2 domains share no primary sequence homology with DH domains.

The crystal structure of ARHGEF3 indicated that the 4–5 loops in the DH domain are flexible and the DH and PH domains interact with each other intramolecularly, thus suggesting that the PH domain rearrangement occurs upon RhoA binding (Murayama et al., 2012). Other RhoA-specific GEFs include PDZ-RhoGEF, leukemia-associated RhoGEF, p115-RhoGEF, and cdc42-specific GEFs. The crystal structure of ARHGEF3 complexed with Rho GTPase will clarify the molecular mechanism underlying its selectivity. Preparation of this complex is currently underway (Murayama et al., 2012).

### 2.2 ARHGEF3 Expression in Tissues and Its Localization in Cells

Most RhoGEFs are widely expressed, and most cell types usually express several RhoGEFs for each GTPase at any given time and under various conditions. Different signals recognize different RhoGEFs based on their domain diversity, which leads to substantially different biological outputs. ARHGEF3 protein expression is found in the heart, kidney, platelets, and macrophages, with the highest levels in skeletal muscles and the brain (Arthur et al., 2002). Notably, ARHGEF3 expression is increased in the early phase of muscle regeneration, suggesting that the expression pattern changes under certain circumstances. The binding between ARHGEF3 and phosphoinositides (PIPs) has been confirmed. PIPs are mostly found in the plasma membrane (PM), and ARHGEF3 binds to PI (4.5) P_2_ and PI (3.5) P_2_ in a PH domain-dependent manner (Singh et al., 2021). In addition, it was observed that PI (4.5) P_2_ binding to ARHGEF3 is essential for its GEF activity, based on an ARHGEF3 mutant (K342E/K348E) lacking PI (4.5) P_2_ binding. However, this mutant retains its binding to PI (3.5) P_2_ (Singh et al., 2021). When EGFP-ARHGEF3 is overexpressed, it localizes to PM as well as the nucleus, probably owing to the presence of a nuclear localization sequence.

### 2.3 Function of ARHGEF3 as a GEF

**FIGURE 2:**
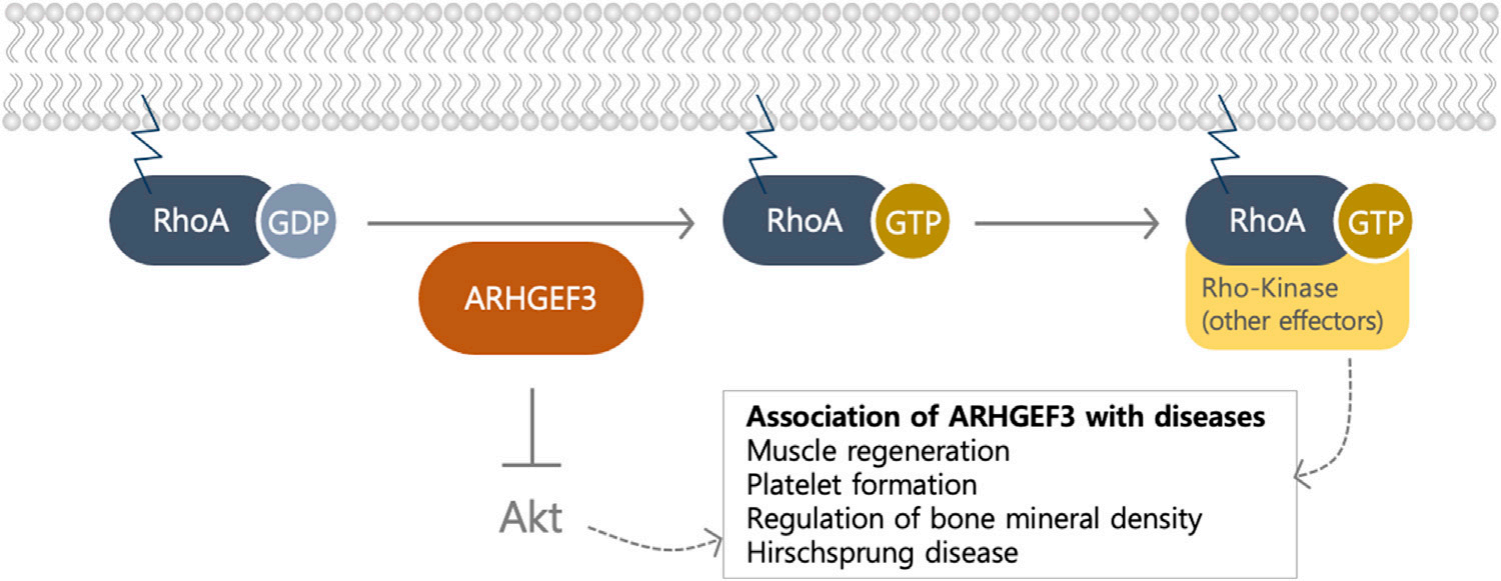
The biological function of ARHGEF3 as a GEF and an Akt inhibitor. ARHGEF3 functions as a GEF to exchange GDP to GTP for RhoA/B and as an Akt inhibitor by binding to mTORC2. The association of ARHGEF3 was revealed via GWAS and knockout organism studies.

ARHGEF3 functions as a RhoGEF, primarily targeting RhoA and RhoB (Arthur et al., 2002) (Figure 2); The role of RhoGEFs is to activate RhoGTPases, the main branch of the Ras superfamily of small (∼21 kDa) GTPases, by catalyzing the exchange of GDP for GTP through stabilization of the nucleotide-free state (Rossman et al., 2005). The bi-molecular cycle between an inactive GDP-bound and an active GTP-bound state facilitates transduction, by interacting with different downstream effectors (Rossman et al., 2005). The DOCK family functions as a GEF for Rac and Cdc42, unlike the Dbl family, which acts on other GTPases, including RhoA (Yang et al., 2009). Since there are approximately four times more RhoGEFs than Rho GTPases (Cote et al., 2005; Yang et al., 2009), multiple GEFs can activate a single GTPase, indicating some redundancy in their functions. At least 25 RhoGEFs can activate the important Rho proteins, RhoA, Rac1, and Cdc42 (Goicoechea et al., 2014). This number is probably an underestimation because the specificities of many RhoGEFs have not yet been thoroughly characterized. ARHGEF3 activates RhoA and RhoB, but not RhoC, although their GTPase sequences are highly conserved (Figure 2). Valine is found at position 43 in RhoA and RhoB, but a bulkier isoleucine residue is found in RhoC (Arthur et al., 2002). The valine positioned at amino acid 43 in the RhoA subfamily determines the substrate specificity of ARHGEF3 (Arthur et al., 2002). It loses extensive solvent-accessible surface area upon binding to the GEF of Dbl proteins, highlighting the role of this residue in the interaction with at least one exchange factor.

RhoGEFs directly link Rho activation to cell surface receptors for various cytokines, growth factors, adhesion molecules, and G protein-coupled receptors. GEFs function immediately upstream of Rho proteins and interact directly with receptors on the cell surface (Bourne et al., 1990; Schmidt and Debant, 2014). RhoA GTPases directly activate Rho kinase, which is essential for stress fiber and focal adhesion formation in response to stimulation by these GTPases (Uehata et al., 1997; Arthur et al., 2002). They are involved in primary cellular functions, such as cell polarity, adhesion, cell motility, growth, and differentiation, and dysregulation of their activities has been associated with disease-related physiological conditions. ARHGEF3 stimulates the formation of stress fibers and focal adhesions in a Rho kinase-dependent manner, and its expression is sufficient for the transformation of NIH 3T3 fibroblasts (Arthur et al., 2002; Snyder et al., 2002). ARHGEF3 regulates intracellular communication together with ARHGEF11 and ARHGEF28 during cell migration, based on shRNA-based screening of 81 Rho-family GEFs known in the human genome (Zaritsky et al., 2017). Accordingly, ARHGEF3 directs long-range communication and rapid signaling by controlling the RhoA/Myosin II pathway, as well as migration directionality, independent of the RhoA-mediated pathway (Zaritsky et al., 2017).

### 2.4 The Noncanonical Function of ARHGEF3 as a mTORC2 Regulator

Several RhoGEF proteins exhibit GEF-independent activity through the N-terminal or PH domains. These noncanonical functions of GEFs have attributed to the modulation of protein-protein interactions through the non-catalytic domain of GEF or through their kinase activity. ARHGEF3 exerts a noncanonical function as an endogenous mTORC2 inhibitor, specifically for Akt (You et al., 2021) (Figure 2). Its noncanonical function occurs via the N-terminal region and the PH domain, as in other GEFs with noncanonical activities. ARHGEF3 was identified as a component of mTORC2, which is one of the two biochemically and functionally distinct mTOR complexes. mTOR is evolutionarily conserved from yeast to mammals and is a serine and threonine protein kinase from the PI3K-related kinase family (Saxton and Sabatini, 2017). In addition to ARHGEF3, mTORC2 consists of rictor (rapamycin-insensitive companion of mTOR), DEPTOR (DEP domain-containing mTOR interacting protein), and the regulatory subunits mSin1 (also known as mitogen-activated protein kinase-associated protein 1 (MAPKAP1)) and Protor1/2 (Saxton and Sabatini, 2017). Khanna et al. performed a yeast two-hybrid screen and found ARHGEF3 to be an mTOR-interacting protein (Khanna et al., 2013). ARHGEF3 specifically binds to mTORC2 but not mTORC1 through its N-terminal region (amino acids 1–125) and the PH domain (amino acids 304–466). Interestingly, ARHGEF3 inhibits mTORC2 kinase activity in a GEF-independent manner. The binding of the N-terminal region of ARHGEF3 to mTORC2 is essential for mTORC2 inhibition, whereas the interaction with the ARHGEF3 PH domain is not sufficient to inhibit mTORC2 activity and could be involved in increasing overall affinity. Since mitogenic stimulation does not change the expression of ARHGEF3 itself or the interaction between ARHGEF3 and mTORC2, ARHGEF3 might control mTORC2 activity through a conformational change or modification of mTORC2 without its physical removal. Notably, its mTORC2-inhibitory role seems to be specific for Akt, a downstream target of mTORC2 (Khanna et al., 2013). To support the noncanonical function of ARHGEF3 as a mTORC2 regulator, ARHGEF3 has been reported to control the regulation of the mTORC2-secreted protein acidic and rich in cysteine (SPARC) axis in idiopathic pulmonary fibrosis (Kamio et al., 2017). The activated histone deacetylase by transforming growth factor β1 decreases ARHGEF3 expression in human fetal lung fibroblast (HFL-1) cells, subsequently leading to increases in mTORC2-Akt activity and the expression of SPARC, one of the regulators of fibrosis formation (Kamio et al., 2017).

## 3 Role of ARHGEF3 in Myogenesis and Muscle Regeneration

The biological function of ARHGEF3 in myogenesis was examined because ARHGEF3 is highly expressed in skeletal muscle among different tissues, and Akt, a specific target of ARHGEF3–mTORC2, is a well-established myogenic factor (Khanna et al., 2013). ARHGEF3 knockdown by introducing lentiviral shRNA for ARHGEF3 augments C2C12 myogenesis, as demonstrated by an increase in the expression of myogenic markers, such as myosin heavy chain and myogenin, as well as in the fusion of myotubes. In contrast, ARHGEF3 overexpression inhibits C2C12 myogenesis in a GEF activity-independent manner. Notably, the N-terminal of ARHGEF3, a binding domain of ARHGEF3 for mTORC2, is essential for its role in myogenesis, implying that regulation of mTORC2 determines the role of ARHGEF3 in myogenesis. Under normal physiological conditions of myogenesis, mTORC2 seems to overcome the inhibitory effect of ARHGEF3 stoichiometrically through an increase in mTOR expression.

The role of ARHGEF3 in *in vitro* myogenesis would be expected to be the same as in an *in vivo* mouse model, in the same biological context. This regulation does not reflect the role of ARHGEF3 in *in vivo* muscle differentiation. Although there were no changes in the phenotype of ARHGEF3 knockout (KO) mouse muscles compared to that of wild-type mouse muscles (You et al., 2021), the size and mass of regenerating fibers and total muscle force were enhanced in tibialis anterior (TA) muscles of ARHGEF3 KO mice, when the TA muscles of ARHGEF3 KO mice were injured by injection with BaCl_2_. Unexpectedly, this enhancement was due to increased autophagic flux through decreased ARHGEF3 GEF activity but not through increased Akt activity. These results imply that the dominant role of ARHGEF3 in RhoA-ROCK regulation is through the control of autophagy during muscle regeneration. Autophagy is an obligatory homeostatic process for degrading unwanted cellular components and providing new protein/energy sources for skeletal muscle differentiation and regeneration (Garcia-Prat et al., 2016; Call et al., 2017; Paolini et al., 2018). Autophagy defects are also related to dynapenia or diminished muscle force without losing muscle mass in the aged muscles (You and Chen, 2021). The depletion of ARHGEF3 restores muscle strength in aged muscles, suggesting a critical role of ARHGEF3 in maintaining the strength of aged muscles. Although Akt is closely related to the regulation of muscle mass and autophagy (Wang et al., 2012; Jaiswal et al., 2019), increased Akt activity is not the primary regulator of autophagic flux or muscle regeneration in ARHGEF3 KO muscles. In contrast, ARHGEF3 expression is increased in the early phase of muscle regeneration despite its effect throughout the process, suggesting an alternative regulatory mechanism of ARHGEF3 GEF activity. In addition, despite a relatively low expression level of ARHGEF3 in muscles, ARHGEF3 functions as a major GEF in muscle regeneration; ARHGEF3 ablation decreases RhoA activity in regenerating muscles (You et al., 2021). Hence, more careful attention is required to infer that XPLN serves as a primary GEF in highly expressed tissue although ARHGEF3 expression level in tissues might help clarify the signaling pathways and biological functions mediated by this protein.

Another topic that will be of considerable interest is the putative role of ARHGEF3 in controlling Akt-involved physiological processes, such as cell survival, glucose metabolism, and other types of cellular differentiation. Indeed, the involvement of ARHGEF3 in metabolism was suggested; the most potent module of expression quantitative trait loci (eQTLs) was found in ARHGEF3 (rs1354034) through integrations with the blood transcriptomic, metabolomic, and genomic profiles from two population-based cohorts (total N = 2,168), including a subset of individuals with matched multi-omic data at 7-years follow-up (Gabriel et al., 2002). This module affects a module related to platelet function. The diverse *trans* eQTL effects of ARHGEF3 (rs1354034) are preserved within the individual over time and may result in extensive interaction with lipoprotein measures (Battle et al., 2014; Jin et al., 2020). This study suggests the potential involvement of ARHGEF3 in metabolic regulation as well as platelet function. Conclusively, future investigation warrants identifying the proper conditions to reduce the redundancy of ARHGEF3 from other GEFs and exploring the putative function of ARHGEF3 in metabolism via stringent conditions such as high-fat diet feeding.

## 4 Association Between ARHGEF3 and Diseases Based on GWAS and Genetic Variation

Several biological actions of ARHGEF3 were estimated based on GWAS. GWASs are the most widely used strategies to identify genetic variations related to phenotypes and disorders in humans (Zou et al., 2017; Eicher et al., 2018). After ARHGEF3 identification as a GEF, several groups predicted its role in several physiological contexts by investigating its genetic variations (Figure 2). eQTL studies link genomic and transcriptome data from the same person, to identify regions that influence mRNA expression which can help annotate GWAS variants by correlating SNPs to changes in gene expression. Integrative analyses based on GWAS are likely to elucidate the pathophysiology of ARHGEF3 variants which improve our understanding of ARHGEF3 biological function.

### 4.1 Role of ARHGEF3 in the Regulation of BMD

The *ARHGEF3* gene is localized within the 3p14-p21 region of the human genome, one of the most replicated QTLs for BMD, implying the potential role of ARHGEF3 in BMD-related diseases (Mullin et al., 2008). Mullin and colleagues investigated sequence diversity within *ARHGEF3* gene using 17 single-nucleotide polymorphisms (SNPs) in a discovery cohort of 769 postmenopausal Caucasian female family members. Among the 17 SNPs, they found a high association between five SNPs and age-adjusted BMD through a family-based association test, suggesting that genetic variation in ARHGEF3 is responsible for determining bone density in Caucasian women. The association between the variation in RhoA and BMD has also been reported (Mullin et al., 2009) to be involved in the potential role of RhoA and RhoA regulators, such as ARHGEF3, in regulating bone density.

In addition, the effect of ARHGEF3 knockdown on the expression of more than 25,000 annotated genes derived from NCBI RefSeq (Build 36.2) was assessed in human osteoblast-like cell lines and osteoclast-like cells derived from a donor (Mullin et al., 2014). ARHGEF3 knockdown markedly decreased the *TNFRSF11B* gene expression, encoding osteoprotegerin in Saos-2 and hFOB 1.19 osteoblast-like cells. As TNFRSF11B expression plays a critical role in the induction of osteoclastogenesis upon exposure to parathyroid hormone, this ARHGEF3–TNFRSF11B association suggests the involvement of ARHGEF3 in this process. However, whether it is related to the GEF function of ARHGEF3 was not evident in this study.

### 4.2 Role of ARHGEF3 in Hirschsprung Disease

Hirschsprung disease (HSCR) is the most common congenital disease of intestinal motility and is characterized by the absence of ganglion cells in the myenteric and submucosal plexuses of the gastrointestinal tract (Gabriel et al., 2002). HSCR is also accepted as a multifactorial genetic model that presents low sex-dependent penetrance, high sibling recurrence risk, high heritability, and interfamilial variation. Hence, genetic factors or gene-gene interaction networks are assumed to be the primary cause of HSCR (Wang et al., 2020). *ARHGEF3* has been identified as an enteric nervous system (ENS) development-related gene through a differential screen for ENS-expressed genes, using RNA from wild-type and RET (RET receptor tyrosine kinase)-mutant (aganglionic) gut tissue and DNA microarray. RET is expressed throughout enteric neurogenesis and is required for normal ENS development. Humans with mutations in the RET locus have Hirschsprung disease, and *ARHGEF3* is located at the RET-dependent susceptibility locus identified at 3p21 (Gabriel et al., 2002). In addition, a recent study reported the association between genetic variants in *ARHGEF3* and HSCR susceptibility in 1,015 subjects (502 HSCR cases and 513 controls) of Han Chinese origin (Wang et al., 2020). Although we did not examine whether ARHGEF3 GEF activity is critical for inducing HSCR in these studies, it was assumed that this activity might be involved in HSCR since the genetic network of ARHGEF3 with the human alpha-catulin gene (CTNNAL1) seems vital in this biological context. CTNNAL1, mapped to the RET-dependent susceptibility locus at 9q31, interacts with Lbc Rho GEF, by providing a scaffold for the latter (Park et al., 2002). The potential regulation of ARHGEF3 in HSCR could be examined because a defect in the migration of enteric neural crest cells is a critical feature of HSCR (Heanue and Pachnis, 2006), and RhoA activity is a critical regulator of cell migration.

### 4.3 Role of ARHGEF3 in Megakaryocyte Development and Platelet Function

In humans, the association between *ARHGEF3* genomic loci and the MPV was identified from the first platelet GWAS, together with WD repeat domain 66, TAO kinase 1, and phosphatidylinositol-4,5-bisphosphate 3-kinase catalytic subunit gamma (Eicher et al., 2018). Another study based on a high-powered meta-analysis of GWAS comprising up to 66,867 individuals of European ancestry showed that a genomic variant at 3p14.3 (SNP rs1354034) exhibits the closest interaction with platelet counts and MPV (Gieger et al., 2011), and *ARHGEF3* was identified as a reliably associated gene with the SNP rs1354034 in genomic loci (Gieger et al., 2011). This SNP was mapped upstream of the crucial *ARHGEF3* isoform in MKs (Zou et al., 2017). ARHGEF3 expression is predominantly enhanced during human and murine MK maturation, supporting the association between this SNP and ARHGEF3 expression. Hence, the involvement of ARHGEF3 in this biological context is expected to be evident in an ARHGEF3 KO animal model.

Silencing ARHGEF3 in *Danio rerio* (Zebrafish) decreases the number of mature erythrocytes and abrogates thrombocyte formation completely (Zou et al., 2017). When the translation of ARHGEF3 was blocked with MO antisense oligonucleotides at an early stage, the ARHGEF3 MO-injected embryos showed normal morphologic development but evident defects in hematopoiesis, especially with microcytic and hypochromic anemia (Serbanovic-Canic et al., 2011). These defects were reproduced in RhoA-depleted embryos. In addition, iron supplementation rescued these defects in ARHGEF3 MO-injected embryos, and transferrin uptake was severely impaired in erythromyeloblastoid cells (K562). These observations suggest that ARHGEF3 regulates transferrin uptake in erythroid cells via its canonical GEF function to activate RhoA.

ARHGEF3 KO mice do not duplicate these defects; Zou and colleagues generated ARHGEF3-KO mice by inserting LacZ reporter cDNA into the endogenous gene to examine the association between ARHGEF3 and MK development and platelet function (Zou et al., 2017). ARHGEF3-KO mice exhibit normal megakaryocyte formation and platelet function, and their platelet counts return to baseline in the same way as that in WT mice in response to acute platelet depletion *in vivo*. The current ARHGEF3-KO mouse model does not mimic genetic variation in humans or has a redundant or compensatory mechanism comprising other RhoGEFs. Hence, future investigations should focus on the role of ARHGEF3 in hematopoiesis and platelet function, as predicted in GWASs.
